# The impact of COVID-19 on UK university students: Understanding the interconnection of issues experienced during lockdown

**DOI:** 10.1177/17577438221104227

**Published:** 2022-06-08

**Authors:** Paul McGivern, Jack Shepherd

**Affiliations:** School of Psychology and Therapeutic Studies, Faculty of Social and Health Sciences, Leeds Trinity University, Horsforth, Leeds, UK; Department of Physics, University of York, York, UK; Department of Biology, 8748 University of York, York, UK

**Keywords:** COVID-19, higher education, university students, student employment, student health, wellbeing, pandemic

## Abstract

The pandemic profoundly disrupted university students’ lives. Many students have parenting and/or caring responsibilities and work part-time jobs. Undergraduate cohorts today are extremely diverse, comprised of people from a wide range of social, ethnic, economical and cultural backgrounds. Research has highlighted the different ways the pandemic has affected the lives of students globally. During lockdown(s) universities responded swiftly to students’ needs enabling them to continue with their studies, though such responses were reactive to targeted needs. Given this, a more granular understanding of the interconnectedness of the issues experienced by UK students during the pandemic is required. This study used conventional Content Analysis to review qualitative responses from 82 participants aged 18+ years. Participants also completed the validated Fear of COVID-19 scale. Three themes: Education, Health, and Quality of Life emerged from the data. The interrelatedness of these themes was highlighted, thus evidencing the complexity of the issues experienced. Overall, Fear of COVID-19 scores were low. These findings have implications for higher educational establishments and wider professional educational bodies moving forward. Whilst higher educational establishments supported students throughout lockdown(s) via targeted responses and interventions, these findings suggest that a more nuanced response to students’ needs is required in future.

## Introduction

SARS-CoV-2, the pathogen which causes COVID-19, was first identified in China in late 2019. COVID-19 is characterized by cough and shortness of breath but has several novel symptoms such as increased blood clotting (Saei et al., 2020). The full range of symptoms is still unclear as efforts continue to try to establish a complete picture of the impact of contracting the disease (Marx, 2021). While most attention was focused on economic and medical impacts, the global pandemic profoundly disrupted the lives of university students (Sahu, 2020). It is therefore important that the higher education sector, and wider professional governing bodies develop an understanding of how issues manifested and how they could be mitigated in future if similar events transpire. Student cohorts today are extremely diverse, comprising people from a wide range of social, ethnic, economical and cultural backgrounds (Jones, 2006) who may have different needs and expectations. Many students work part-time jobs, (Tessema et al., 2014) have parenting and/or caring responsibilities and financial dependents/responsibilities (Stone and O’Shea, 2019). Reflecting this, in recent years, universities have responded positively by developing an increased focus on pastoral support, student wellbeing services, and formal social events in addition to the delivery of course content and the more traditional aspects of the student experience (Segaren, 2019). Moreover, universities have been instrumental in raising awareness on contemporary social issues and promoting student voice as they prepare students for their chosen careers, helping them to become educated, empathetic, supportive and considered members of their chosen communities and workplaces. However, the disruption caused by the global pandemic meant that radical changes needed to be implemented to enable the continuation of higher education studies. Students had to switch on an online mode of learning (Irawan et al., 2020) and many students were either forced to isolate in their student-accommodation or forced to leave, which subsequently caused further issues regarding their personal lives.

The United Kingdom (UK) Office for National Statistics (ONS) recently reported useful statistics in relation to student experiences during lockdown, with 29% of students reporting dissatisfaction regarding their student experience. Moreover, 65% reported issues in relation to their accommodation, and a decrease in overall life satisfaction (Zimmermann et al., 2020). The global disruption caused by the pandemic has naturally led to increased uncertainty across many sectors in the market economy including the job market (Baker et al., 2020), which also impacts on students. Additionally, both public and private sector organisations have dramatically changed the ways in which they work with many staff working remotely, and/or conducting part or all of their duties online or in new ways. It is unsurprising that the dramatic impact that the global pandemic had on both education and employment prospects triggered broader peripheral issues in students’ lives; specifically, how social isolation impeded students’ ability to maintain relationships with family, friends and loved ones (Long et al., 2021). Taken together, it is reasonable to infer that many students will have become fearful of contracting COVID-19 for fear of becoming ill themselves or passing it on to others (Rodríguez-Hidalgo et al., 2020).

During lockdown(s), university students had to endure a series of largely untested major changes, which impacted on the ways in which they would continue learning, and how and where that would take place. At the time of writing, there is a growing body of research focused on developing an improved understanding of how the global pandemic and multiple lockdowns impacted on a variety of demographics across different countries and nations (Dinh and Nguyen, 2020; Giusti et al., 2020; Odriozola-González et al., 2020; Wang and Zhao, 2020; Zimmermann et al., 2020). However, a more refined understanding of the impact of the pandemic on UK university students would further benefit professionals and policymakers in higher education spheres. While existing UK-based quantitative research has been effective in developing a broad-stroke understanding of the issues experienced by student demographics, Tremblay et al. (2021) highlight the value of qualitative research as a particularly beneficial methodology to help ascertain a more granular understanding of the issues experienced in relation to COVID-19. More broadly, such inductive, exploratory approaches can produce valid and insightful findings (Reiter, 2017). The aim of the present study was therefore to elucidate in more detail, the experiences of UK university students during lockdown(s) and the interrelatedness of their concerns.

## Methods

The present study employed conventional qualitative Content Analysis (CA) (Hsieh and Shannon, 2005) to explore the impact of COVID-19 on UK university students. Using CA enables researchers to avoid predetermined concepts and instead allows categories and themes to emerge naturally from the dataset. Such an approach was appropriate given that research and theory surrounding the impact of the pandemic on UK university students is – at the time of writing – a growing area of research. Participants were recruited via convenience sampling from a university in the Northeast of England. All participants were current students at either undergraduate or postgraduate level. The collected data were analysed using the CA approach considering both implicit and explicit content. A series of codes were developed and subsequently categorised; this process led to the identification of three key themes (see Table 1).

**Table 1.** Themes and categories.

Theme	Categories
1. Education	Inability to study effectively
	Lack of value for money
	Impact on grades
	Reduction in teaching quality
	Lack of support
2. Health	Physical health
	Mental health
3. Quality of life	Issues gaining employment and broader financial concerns
	Socialisation and isolation
	Maintaining relationships
	Poorer living arrangements/Conditions

### Ethical considerations

All data were collected using the online survey tool *Qualtrics*. Each participant received a URL to access a survey, which was administered via university email addresses to ensure that the sample comprised only current university students. The opening screen of the survey provided details of the aims of the study inclusive of ethical implications. Each participant provided a pseudonym, and consent was obtained electronically when participants agreed to take part in the study (if a participant selected ‘no’ when providing consent to take part, the survey navigated to the final screen and thanked participants for their interest). Upon gaining consent, participants completed the survey, which included opportunities to respond to open-ended questions. Participants did not have to respond to any questions that they did not want to respond to and could withdraw from the study at any time during and up to 2 weeks after completing the study (zero participants met this criterion). Upon completion of the study, participants were debriefed and thanked for their participation. The study was approved by the local university ethics committee.

## Results

A total of 82 participants (Males = 7, Females = 75) took part in the study. The mean age of the sample was 21.64 years (SD = 5.30). Fear of COVID-19 was measured as part of the survey using the Fear of COVID-19 Scale (Ahorsu et al., 2020). A Cronbach’s Alpha (α = 0.89) measure reported a high level of internal consistency. The Fear of COVID-19 rated scale captures scores on a 5-point Likert scale with total scores ranging from 7–35. Higher scores represent greater levels of fear. In total, 77 of the 82 participants completed the Fear of COVID-19 scale. Of those 77 participants, overall levels of fear were low (M = 10.8, SD = 3.45). Therefore, no further analyses were conducted based on the Fear of COVID-19 scale measure. Qualitative analysis revealed three themes of: Education, Health, and Quality of Life each with a range of sub-categories (see Table 1 below).

## 1. Education

Of the sample, 62 participants reported that the pandemic had negatively impacted their education in a range of ways. The theme of Education comprises five categories: Inability to study effectively, lack of value for money, impact on grades, reduction in teaching quality and lack of support.

### Inability to study effectively

A total of 36 participants referred to their inability to study effectively during lockdown. Noisy and unsuitable accommodation and/or environments were frequently reported, as many students have parental duties. Additional problems centred around technical issues, such as poor Wi-Fi connectivity, difficulty accessing appropriate resources and excessive screen time. This is also impacted on motivation (or lack thereof) to attempt to engage with their studies.

### Lack of value for money

Ten participants made explicit reference to the lack of value for money that they had paid for their education. Comments such as ‘I am not receiving the education I paid 9k for’ and ‘…you are not getting an education worth nine-thousand pounds’ highlighted disdain felt by many students in relation to the quality of materials and support that they felt they were receiving.

### Impact on grades

Eight participants referred to the negative impact that COVID-19 and lockdown had had on their grades. These students felt that the switch to online learning coupled with poorly organised materials resulted in lower ‘… grades, meaning that I haven’t achieved my best’. Another student reported that their ‘… grades had dropped 10% since the first lockdown’ which was also linked to general issues with working environments.

### Reduction in teaching quality

Eighteen participants reported that the changes regarding the delivery of materials had impacted negatively on their overall quality. Students reported ‘dips’ in standards and that online learning was insufficient by comparison to face-to-face learning due to the ‘disconnect’ between lecturer and student(s). Many students reported feelings of frustration regarding institutions’ outlook on online learning, such that it was ‘unfair to say [that] we are getting the same quality of education we would be if we were on-campus’.

### Lack of support

Sixteen participants reported that they felt that general support with their studies had noticeably reduced. Issues with communication and an inability to work on-campus meant that support was perceived to be less visible, with students reporting that they felt like they were ‘…just expected to get on with their degree’ and that ‘support day-to-day was unsuitable’.

## 2. Health

A total of 50 participants reported that COVID-19 had impacted on either one or both aspects of their physical and mental health. Prominent were reports of increased depression, anxiety, stress and worry, alongside reductions in self-esteem. The negative impacts on physical health manifested in symptoms related to COVID-19.

### Physical health

Eight participants reported that the pandemic had a detrimental impact on their physical health. Disruption to daily routines had led to poor diet, a lack of sleep, and a limited ability to be able to exercise and study effectively. Participants also reported that COVID-19 had exacerbated pre-existing medical conditions causing further psychical health difficulties.

### Mental health

The negative impact of COVID-19 on participants’ mental health was the most prevalent finding in the dataset with 43 participants reporting such issues. Reports of anxiety, stress, depression and loneliness had been triggered by a range of issues regarding the disruption to students' lives. Many reported more generally that their mental health had deteriorated, which had a knock-on effect regarding motivation to study and a general lack of energy.

## 3. Quality of life

A total of 53 participants reported that COVID-19 had impacted negatively on their overall quality of life. This theme comprised five categories: Employment Worries, Socialisation and Isolation, Difficulties Maintaining Relationships and Poorer Living Arrangements/Conditions.

### Issues gaining employment and broader financial concerns

Of the sample, 40 participants reported concerns directly related to employment and financial worries. Many students reported worries regarding their ability to gain employment upon completion of their studies. Fears related to increased competition for limited opportunities were commonly reported (e.g., ‘Trying to find a job is really difficult as there’s lots of competition’.). Worries concerning the outcome of a lower overall degree classification due to COVID-19 restrictions were also linked to employment concerns. Others reported the impact that employment worries had had on broader life-plans, making comments such as, ‘There’s so much uncertainty in the job market that it is difficult to know the right move to make’ and ‘I am losing my independence [because of] having to move back home to ensure financial security’.

### Socialisation and isolation

Issues related to a lack of socialisation and prolonged isolation featured among 23 participants. Being unable to see friends and family led to reports of demoralisation, and a lack of motivation to engage with daily activities, including their studies. Many reported that they felt that support networks had been lost leading to negative lifestyle changes (e.g. ‘I am also extremely active and social [but] now I am a very lazy, lonely and quiet person. I just have no energy’.)

### Difficulties maintaining relationships

Twenty-six participants reported difficulty maintaining various relationships, including romantic relations, friendships, and family relationships. A small proportion of participants reported the loss of loved ones to COVID-19, while many other students reported that they felt that they were not getting a ‘proper’ university experience due to weakened connections with family and friends. Many reported the difficulties felt in assessing the potential benefit of visiting others versus the risk of passing on the virus. A proportion of participants also reported issues generated due to sustained periods of isolation with others, which in turn had a detrimental impact on existing relationships.

### Poorer living arrangements/conditions

Finally, nine participants reported that the pandemic had a significant impact on their living conditions. It is important to note such issues were directly related to their student status insofar as issues were linked to student-accommodation and/or house-sharing, which are both common forms of residency among UK student demographics. Some participants reported having to continue to pay for accommodation despite being unable to live in it and one participant reported that they became homeless. Again, disruptions to, and/or improper living arrangements also had a negative effect on other aspects of students’ personal lives, mental health and ability to study effectively.

## Discussion

Previous studies have highlighted the range of issues experienced by university students during UK lockdowns. This study aimed to contribute to this body of research to assist in better understanding the complexities of how such issues manifested. Specifically, the study aimed to develop an understanding of the interconnectedness of such issues. The findings from the present study align with the general findings from the ONS (Zimmermann et al., 2020) but improve our understanding of the interrelatedness of the themes emphasised within. Specifically, whilst the three core themes evidence pertinent issues in their own right in terms of their negative impact on students’ lives, it is more important to acknowledge that these issues, and the subthemes that they comprise, are inextricably linked. Moreover, these findings illustrate a cross-subtheme interconnectedness whereby issues occurring in one aspect/theme of a student’s life impact on, and potentially exacerbate the negative experiences in other aspects of their life. The present study thus highlights the complexity of the issues experienced, due to the reciprocal negative impact that the subthemes across all three core areas have on each other (i.e. a compounded impact on students’ lives).

Interestingly, reported fear of COVID-19 was low across the sample. When examined in conjunction with the themes highlighted within, it is reasonable to infer that students clearly have concerns about the impact that COVID-19 can have on their lives more broadly but are generally less fearful of the virus itself. Low levels of fear may be explained by the fact that early reports stated that young people were more likely to only experience mild symptoms of the virus (Yuki et al., 2020). However, though this is still the case, recent reports indicate that Long COVID is a concern for all individuals, including young adults, with approximately 134,000 people aged 17–25 years old having self-reported long COVID symptoms (Vagnoni, 2021). Long COVID is the term used to describe enduring symptoms such as fatigue, breathlessness, chest pain, and problems with concentration that remain after the virus has left the body (Aiyegbusi et al., 2021), and is an aspect of the virus that science is yet to fully understand (Brown and O’Brien, 2021). Given that this is still an emerging area of research, future studies should continue to explore the evolving relationships between fear of COVID-19 and the wider impact of COVID-19 on students’ lives.

At the time of writing, the UK, along with the rest of the world face further potential lockdowns as new more dangerous variants of the virus continue to emerge (The World Health Organization, 2021). Furthermore, as we emerge from the pandemic with a view to moving back to a more normal way of living, organisations and service-providers in both private and public sectors are working to establish optimal frameworks that are more resilient to any potential future radical changes in day-to-day operations. Higher Educational institutions have learned a lot from the issues caused by the pandemic. Both staff (Rasiah et al., 2020) and students (Dos Santos, 2020) sacrificed a great deal in order for the provision of education to continue but also developed new skills along the way, specifically, the digital upskilling of staff and students (Snelling, 2022). Naturally, responses to students’ issues such as accommodation, learning, and wellbeing were not always as optimal given that they were often reactive to everchanging conditions and situations, and targeted to specific needs. Responses to student issues – particularly those discussed herein – were often addressed in isolation and in alignment with government policy. Therefore, changes to students’ mode of learning, living conditions, work–life balance, and the subsequent impact on health occurred incrementally. As a result, whilst many issues experienced by students may appear to have been resolved when viewed in isolation (e.g. provision of online learning materials and personalised online support) the findings of the present study suggest that the collective outlook for students during lockdown(s) was even more problematic and challenging than perhaps thought.

The authors acknowledge the potential limitation that the study comprised data collected from a single UK institution. However, the success of widening participation initiatives across UK universities in recent years (e.g. Hayes, 2019; Jones, 2006), means that UK student cohorts share similarities regarding age, gender, ethnicity and socioeconomic status. Furthermore, with regards to the pandemic, it is well-documented that UK universities took a unified approach to enable students to continue to engage with their studies (The Quality Assurance Agency for Higher Education, 2020) thus experiencing the same challenges and difficulties along the way. Taken together, it is reasonable to infer that the experiences documented herein largely resonate with student experiences across the UK.

## Conclusions

It is important that educational professionals remain mindful of the broader facets of students’ lives; namely family, relationships, financial dependents and financial responsibilities (Stone and O’Shea, 2019; Tessema et al., 2014). Moving forward, the findings of the present study suggest that higher education establishments require a more nuanced response to students’ needs when working under future similar challenging conditions. A more comprehensive and collective response to students’ needs may serve to improve students’ circumstances and reduce subsequent negative student experiences, particularly given the diverse nature of student cohorts today.
